# HIV knowledge and risk behaviors among drug users in three Vietnamese mountainous provinces

**DOI:** 10.1186/s13011-019-0191-8

**Published:** 2019-01-15

**Authors:** Tam Minh Thi Nguyen, Bach Xuan Tran, Mercedes Fleming, Manh Duc Pham, Long Thanh Nguyen, Anh Lan Thi Nguyen, Huong Thi Le, Thang Huu Nguyen, Van Hai Hoang, Xuan Thanh Thi Le, Quan Hoang Vuong, Manh Tung Ho, Van Nhue Dam, Thu Trang Vuong, Vu Nguyen, Huong Lan Thi Nguyen, Huyen Phuc Do, Phuong Linh Doan, Hai Hong Nguyen, Carl A. Latkin, Cyrus S. H. Ho, Roger C. M. Ho

**Affiliations:** 1 Vietnam Authority of HIV/AIDS Control, Ministry of Health, Hanoi, Vietnam; 2 Institute for Preventive Medicine and Public Health, Hanoi Medical University, Hanoi, Vietnam; 3 Bloomberg School of Public Health, Johns Hopkins University, Baltimore, MD USA; 4 Vietnam Young Physician Association, Hanoi, Vietnam; 5 University College Dublin, Belfield, Dublin, Ireland; 6 National Institute of Hygiene and Epidemiology, Hanoi, Vietnam; 7 Centre for Interdisciplinary Social Research, Phenikaa University, Hanoi, Vietnam; 8 Université Libre de Bruxelles, B-1050 Brussels, Belgium; 9 Institute of Philosophy, Vietnam Academy of Social Sciences, Hanoi, Vietnam; 10 National Economics University, Hanoi, Vietnam; 11 Sciences Po Paris, Campus de Dijon, 21000 Dijon, France; 12 Department of Neurosurgery Spine-Surgery, Hanoi Medical University Hospital, Hanoi, Vietnam; 13 Institute for Global Health Innovations, Duy Tan University, Da Nang, Vietnam; 14 Center of Excellence in Evidence-based Medicine, Nguyen Tat Thanh University, Ho Chi Minh city, Vietnam; 15 Department of Psychological Medicine, National University Hospital, Singapore, Singapore; 16 Department of Psychological Medicine, Yong Loo Lin School of Medicine, National University of Singapore, Singapore, Singapore; 17 Center of Excellence in Behavioral Medicine, Nguyen Tat Thanh University, Ho Chi Minh City, Singapore

**Keywords:** Methadone maintenance, HIV, Mountainous Vietnam, Knowledge, Attitudes, Practices

## Abstract

**Background:**

Globally, people who inject drugs are highly vulnerable to HIV transmission. Methadone maintenance treatment (MMT) programs are one of the most cost-effective mechanisms to substitute opioid use and improve the quality of life of patients. Since the coverage of MMT is still limited and even for those patients who are treated, improving their knowledge on HIV and maintaining healthy behaviors are key to maximizing the outcomes of HIV harm reduction programs. This study examined the knowledge on HIV, perceived risk and HIV testing among drug users accessing methadone maintenance services in three Vietnamese mountainous areas.

**Methods:**

A cross-sectional study of 300 people enrolling for MMT services in three provinces in Vietnam was conducted. The factors associated with the knowledge, attitudes, and practices of respondents about HIV/AIDS were exploited using multivariable logistic model.

**Results:**

Of the 300-people surveyed, 99% knew of HIV and 60.6% were identified as having good knowledge. While 75.2% identified that injecting drugs was a risk factor for HIV, 52.2% thought they were not at risk of HIV mainly as they did not share needles. 92.6% had undergone HIV testing with 17.4% being positive, a number which was significantly lower than Vietnam’s national average for people who inject drugs. Age, ethnicity and education were associated with knowledge of HIV while ART treatment was linked to self-assessed HIV status.

**Conclusions:**

This study sheds new light on the knowledge attitudes and practices of people who inject drugs, particularly males in mountainous areas of Vietnam regarding HIV prevention. Overall, knowledge was good with most conducting safe practices towards transmission. Enhanced education and targeting of minority groups could help in increasing the numbers receiving MMT and HIV services.

## Background

Globally, people who inject drugs (PWID) remain a highly vulnerable group when it comes to HIV infections [1]. Of the estimated 13–16 million people living worldwide who inject drugs, it is though that up to 1.7 million are living with HIV [2, 3]. Injecting drug practices account for up to 10% of new HIV infections and when Africa is excluded, 30% [4, 5]. Drug injection as a risk factor in contracting HIV was implicated in causing 2.0 million Disability-Adjusted-Life Years (DALYs) in 2010 [3]. As well as this, HIV is the major cause of morbidity and mortality among PWID [6]. This demonstrates the significant economic burden that HIV infections have especially when in addition to the drug use burden, which in the United States in 2007 was approximately $193 billion [7]. The burden of HIV infections among PWIDs is not solely economic however. The individual and social impact of both is significant. As both groups are typically exposed to stigma and ostracized from communities, obtaining work and playing a “normal” role in society can be difficult. Being both a PWID and HIV positive can potentiate these effects. This can have substantial consequences for patients’ mental health.

One of the most cost effective health care interventions for PWID is the introduction of methadone maintenance treatments (MMT) to assist people in withdrawing from using opioid drugs [8, 9]. As well as being cost-effective, MMT has been shown in several studies in Malaysia, Taiwan and China to be efficacious in reducing the needs of PWID to use other drugs and in the improvement of socioeconomic factors for these people [10–12]. The positive effects of MMT have been seen when applied to HIV-positive PWID as well. Globally, MMT has been demonstrated to have positive effects on HIV patients’ treatment and risk behavior patterns as well as in the prevention of new HIV infections [13–15]. As reviewed by Karki et al. patients who initiate MMT programs globally have been shown to have reduced risk of HIV, improved HIV prevention behaviors and are more likely to engage with HIV testing [14].

Since 2008, Vietnam has introduced, and rapidly developed, an MMT plan to reduce the number of PWID within the country and in turn, to help to decrease HIV transmission [16]. Currently there are 251 MMT centers across Vietnam catering to about 46,000 patients [17]. As well as this, HIV treatment services have been rapidly improved to include 364 centers [18]. In Vietnam, currently about 260,000 people have been diagnosed with HIV with rates remaining stable over the last 3 years [19]. In some provinces up to 30% of these HIV patients are PWID [20], highlighting the impact that intravenous drug use has on HIV transmission. In previous studies, changes to drug use behavior due to MMT in Vietnam have been associated with reduced risk behaviors and improved quality of life among patients [21]. MMT services are now seen as a key mechanism for accessing the hidden drug using population in Vietnam as funding for peer-to-peer outreach declines [22, 23]. In addition, by accessing MMT programs, patients can be further referred to other HIV/AIDS or general health services in a timely manner. However, the current coverage of MMT services is still limited, especially in mountainous areas. In addition, even for those patients who were treated with the methadone medication, improving their knowledge of HIV/AIDS and risk behaviors is key to maximizing the impact of harm reduction programs.

Expanding MMT services as well as other harm reduction activities in mountainous areas, has been very challenging given the geographical barriers, patients’ mobility and there being higher rates of health problems and risk behaviors. However, data regarding the characteristics of the target population accessing MMT in this setting is very limited. This study examined the knowledge on HIV, perceived risk and HIV testing among drug users accessing methadone maintenance services in three Vietnamese mountainous areas. The findings of this study may inform the expansion strategies of MMT as well as assist in designing other necessary harm reduction interventions for drug users in this setting.

## Methods

### Study setting

A cross-sectional study was conducted in December 2014 in three mountainous provinces Dien Bien, Lai Chau and Yen Bai. Clinics providing HIV/AIDS services which met the following criteria were chosen: 1) Having integrated MMT services; 2) being in an urban or rural area; and 3) providing service at the provincial and district levels of the Vietnamese healthcare system. Patients who registered at Provincial AIDS Centers in Dien Bien, Lai Chau and Yen Bai City; and District Health Centers in Thanh Xuong, Phong Tho and Nghia Lo District for services such as HIV testing or treatment were interviewed. While patients had availed of HIV/AIDS services this did not necessarily mean they needed HIV treatment or were HIV positive.

We invited patients who fulfilled the eligibility criteria of: i) using MMT services at one of the chosen clinics; ii) being 18 years old or older; iii) being able to answer the structured questionnaire. We used a consecutive sampling approach to recruit participants. We interviewed 300 drug users, all of whom met the criteria and agreed to join in the study. All (100%) of enrolled participants were male, due to the fact that our selected clinics consisted of only male patients.

### Measures and instruments

Face-to-face interviews of 15-min duration using structured questionnaires were conducted to collect data from the participants. Data collectors were post-graduate students from Hanoi Medical University who were trained in conducting community survey prior to this study. The questionnaire was piloted in 10 MMT patients, revised based on their feedbacks and implemented widely after that.

We asked drug users about their knowledge, attitudes and practices of HIV prevention and treatment. The knowledge surveyed included: 1) HIV/AIDS transmission; 2) susceptibility to HIV infection; and 3) HIV prevention according to 5 questions set by the Vietnam Ministry of Health in 2007. The respondents were asked to answer 5 True/False questions. If drug users answered all questions correctly, they had good knowledge and if they answered incorrectly, they had poor knowledge. The internal consistency was low with the Cronbachs’ alpha = 0.5074.

The attitudes surveyed included: 1) self-assessed HIV status (meaning the HIV status participants felt they were – HIV positive or negative, or as having their HIV viral load suppressed – but without confirmatory medical testing); 2) reasons for risk of HIV/AIDS infection; and 3) reasons for no risk of HIV/AIDS infection. The practices surveyed included: 1) using HIV testing and counselling services before beginning MMT; 2) provider-initiated testing and counseling; 3) HIV Testing; and 4) HIV Testing result.

In addition, we also asked participants about their socio-demographic status (age, ethnicity, education, marital status, occupation), health status (any health problem in the last 30 days, HBV, HCV, opportunistic infections and Antiretroviral Therapy history), and their duration of using illicit drugs.

### Statistical analysis

We analyzed data by using STATA software version 12.0 (StataCorp. LP, College Station, TX, USA). First, we described the frequency (n) and percentage occurrence (%) of variables. To identify associated factors with good knowledge, levels of self-assessed HIV status and the HIV testing uptake among drug users, we used a multivariable logistic regression model. Potential associated factors with knowledge about HIV/AIDS included socio-demographic characteristics (age, ethnic, education, marital status, occupation), health problems in the last 30 days, currently enrolling in antiretroviral therapy and duration of drug use. Meanwhile, along with this pool of variables, we also tested the association between knowledge about HIV/AIDS and perceived HIV status and HIV testing uptake. A stepwise forward strategy including variables which have a *p*-value less than 0.1 was used as the reduced model to exclude variables with p-value greater than 0.2.

## Results

A total of 300 participants enrolled in the study. The demographic characteristics of these patients are described in Table 1. All participants were male due to the centers that were chosen. Most participants were middle aged, with 36% being 30–39 years old and 35.7% being 40–49. Kinh and Tay were the two most common ethnicities. The minority of participants (8.7%) had an education level higher than high school. Most participants were freelancers and lived with a spouse/partner. Very few had health problems in the last 30 days (11.3%) and only 10.7% of all participants were receiving ART. Duration of drug use varied however most had used for more than 2 years.

**Table 1:** Demographic characteristics of respondents

Characteristics	*n*	%
Age group		
< 30	51	17.0
30–39	108	36.0
40–49	107	35.7
≥ 50	34	11.3
Ethnicity		
Kinh	176	58.7
Tay	14	4.7
Thai	81	27.0
Others	29	9.7
Education		
< High school	168	56.2
High school	105	35.1
> High school	26	8.7
Marital status		
Single	79	26.3
Live with spouse/partner	177	59.0
Widow/Divorced	44	14.7
Occupation		
Unemployment	24	8.0
Freelancer	249	83.0
Worker/White-collar worker	9	3.0
Others	18	6.0
Having any health problem in the last 30 days		
No	266	88.7
Yes	34	11.3
Antiretroviral therapy (ART)	32	10.7
Duration of drug use		
< 2 years	13	4.8
2–5 years	79	29.3
5–10 years	90	33.3
10–20 years	68	25.2
> 20 years	20	7.4

Knowledge about HIV prevention and treatment among drug users is presented in Table 2. Almost all participants had heard of HIV. The majority (83.3%) identified sharing needles and syringes as causing HIV transmission along with unsafe blood transfusions (67.9%) and unprotected sex (87.3%). More than half of the patients did not know that HIV could pass from mother to child. 94.7% identified that PWID were susceptible to HIV. The majority thought that faithfulness to partners and correct condom use could prevent/reduce HIV infection risk. Interestingly, the majority thought HIV could be spread by mosquitos (86.6%) or by eating with an infected person (91.6%). Despite this, 60.3% said their HIV knowledge was good.

**Table 2:** Knowledge about HIV prevention and treatment among drug users

Characteristics	*n*	%
Heard about HIV/AIDS		
No	3	1.0
Yes	297	99.0
HIV/AIDS transmission		
Blood Transfusion Unsafety	203	67.9
Sharing needles and syringes	249	83.3
Transmissible from mother to child	143	47.8
Unprotected sex	261	87.3
Susceptibility to HIV infection		
People who inject drugs	283	94.7
Sex workers	229	76.9
Long distance highway drivers	18	6.0
Multiple sex partners	89	29.9
Faithfulness to partners: a means to prevent HIV infection		
False	36	12.2
True	260	87.8
The correct use of the condom reduces the risk of HIV infection		
False	10	3.3
True	289	96.7
A healthy or healthy-looking person can be HIV-positive		
False	69	23.2
True	229	76.9
Mosquitoes/insect can transmit HIV		
False	40	13.4
True	258	86.6
HIV infection is transmitted through eating with a person with HIV-infected		
False	25	8.4
True	273	91.6
Knowledge about HIV/AIDS		
Not good	117	39.7
Good	178	60.3

Table 3 describes the attitudes about HIV prevention and treatment among drug users in Vietnam. Over a half of study participants reported that they were self-perceived as being at no risk of HIV (52.2%) while 75.2% identified drug injecting as a reason for risk of HIV. The majority of participants identified not sharing needles (66.7%) as a reason for no risk of HIV. Not having sex with female sex workers, using condoms and being faithful were other common reasons for no risk of HIV infection.

**Table 3:** Attitudes about HIV prevention and treatment among drug users

Characteristics	*n*	%
Self-assessed HIV status		
No risk	143	52.2
Low risk	67	24.5
High risk	64	23.4
Reasons for risk of HIV infection		
Many partners	18	14.1
Sex without using condom	17	13.3
Drug-injection	97	75.2
Receiving blood transfusions	6	4.7
Reasons for no risk of HIV infection		
Being faithful	65	35.0
Using condom	39	21.0
Not sharing needles	124	66.7
Not having friends with HIV infection	11	5.9
Not having anal sex	18	9.7
Not having sex with female sex workers	53	28.5
Not receiving blood transfusions	7	3.8

Patients’ HIV testing histories are described in Table 4. Most patients (82.2%) had availed of HIV testing and counselling services prior to MMT and for 60.2%, this took place at provincial centers for HIV/AIDS prevention. 92.6% had had a HIV test and for 76.5% of these, the test was negative.

**Table 4:** Practices for HIV prevention and treatment among drug users

Characteristics	*n*	%
Before beginning MMT, using HIV Testing and Counselling Services		
No	53	17.8
Yes	245	82.2
Provider-initiated testing and counseling		
Commune Health Stations	24	12.9
District health bureau	39	21.0
Provincial Center for HIV/AIDS Prevention	112	60.2
Others	11	5.9
HIV Testing		
No	22	7.4
Yes	276	92.6
HIV Testing result		
Positive	48	17.4
Negative	211	76.5
N/A	17	6.2

Table 5 examines factors associated to the knowledge, attitudes and practices of HIV prevention and treatment among male PWID. Knowledge of HIV was negatively associated with participants aged 30–39, or those who were of Thai or other ethnic background compared to Kinh. The likelihood of knowing about HIV/AIDS was greater among those who were high school educated than those with less than high school. Although it was just “borderline” statistically significant, it is important to notice that patients who had 5–10 year of drug abuse were less likely to take HIV tests than those with less than 2 years drug use history.

**Table 5:** Associated factors related to knowledge, attitudes and practices of HIV prevention and treatment among drug users

Characteristics	Knowledge on HIV/AIDS		Perceived HIV status		HIV Testing	
	OR	95% CI	OR	95% CI	OR	95% CI
Age groups (< 30 - ref)						
30–39	0.39***	0.22; 0.69				
Ethnicity (Kinh - ref)						
Tay			0.43	0.12; 1.55		
Thai	0.47**	0.25; 0.87			0.49	0.19; 1.30
Others	0.35**	0.13; 0.93				
Education (<High school - ref)						
High school	2.76***	1.51; 5.05				
> High school	2.3	0.78; 6.76				
Occupation (Unemployment - ref)						
Worker/ White-collar worker			2.92	0.66; 12.91		
Duration of drugs use (< 2 years - ref)						
5–10 years					0.45*	0.18; 1.16
> 20 years	0.41*	0.15; 1.14	2.02	0.79; 5.20		

***p < 0.01, **p < 0.05, *p < 0.1; − ref Comparison group

## Discussion

In this sample of male PWID prior to receiving MMT, we found that a large proportion of them had a good knowledge on the risks of HIV transmission. However, misconceptions about the forms of transmission were still seen as well as there being a lack of appreciation of the magnitude of unprotected sex for HIV transmission. Scaling up HIV testing and opioid dependence treatment services as well as expanding mass media campaigns are recommended in these disadvantaged settings.

This study contributes to the body of evidence on the HIV-related knowledge, attitudes and practices (KAP) for PWID commencing MMT in mountainous areas. This is important as PWID are at high risk of contracting HIV due to unsafe behaviors, especially in Vietnam where the HIV epidemic is concentrated among PWID. In addition, implementing interventions in disadvantaged settings are more challenging given the poor accessibility to respondents. By focusing on mountainous regions where MMT and HIV services are less prevalent, information can be gathered about how to enhance HIV prevention and minimize risk behavior. We found that misconceptions in some routes of HIV transmission still exist in this setting. Most patients knew that needle sharing leads to HIV transmission, however, mosquitoes and sharing food were also thought of modes of transmission. Most identified their knowledge of HIV as being good but also indicated that they had no risk of HIV. Before commencing MMT, 92.6% had taken a HIV test with 17.4% being positive. Age, ethnicity and education level were identified as potentially being associated with knowledge of HIV/AIDS.

Overall, the level of ‘good’ knowledge about HIV/AIDS (60.3%) was similar to other studies globally [24–26]. Most knew of the major modes of transmission, such as unsafe blood transfusions, unprotected sex and sharing needles and syringes, however only a minority knew that it could be passed from mother to child. Participants also identified that PWID and female sex workers were susceptible to HIV however only a third identified multiple sex partners as a risk. Faithfulness and condom use were known as methods of preventing transmission whereas, interestingly, most participants thought that mosquitoes and sharing food could transmit HIV. This result agreed with a similar study on knowledge of HIV in Iran, in which mosquitoes were identified as a vector of transmission [27]. In other studies, such as in Brazil and Cameroon, knowledge of transmission routes was significantly better, with only the minority in both cases reporting mosquitoes and food sharing as a mechanism for transmission [25, 26]. These differences in knowledge level may be attributed to the mountainous region in which this study was conducted. In other studies, urban and metropolitan areas were used. This could lead to a more educated sample, who had greater access to health service being chosen in the past. In Vietnam, there are differences in the level of educational services provided between provinces with the central, city regions having more higher-education opportunities [28]. In this study, the likelihood of good HIV/AIDS knowledge was higher among participants who were high school educated, demonstrating the impact that education can have for PWID. This agrees with international and Vietnamese data where education level and HIV knowledge are positively associated [29–31]. Increasing the access of PWID to further education could benefit their knowledge of HIV with an aim to decreasing the risk of transmission.

Smaller ethnic groups in mountainous areas were less likely to have good knowledge of HIV/AIDS compared to the majority group, Kinh. In other studies regarding health service utilization in mountainous areas of Vietnam, Kinh people were also shown to be the majority ethnic background utilizing services [32] despite minority groups being prevalent in these regions [33]. Malqvist et al. has suggested that discrimination in health service utilization still exists in Vietnam [34]. This finding indicates a cohort of PWID may have poor health service utilization and therefore need to be targeted to improve HIV knowledge. As ethnic groups are spread over a wide geographical, mountainous area, difficulty in getting to health service locations, both for MMT and HIV may be substantial and lead to decreased knowledge of HIV among these participants.

The majority of participants in our study identified that they were at no risk of HIV/AIDS, despite being PWID and having identified drug injection and PWID as being at higher risk. Reasons for this lack of risk varied however the most common was being faithful in sexual relationships, not sharing needles and not having sex with female sex workers. Only 21% reported to using condoms, a key practice in reducing the risk of HIV transmission [35]. Similar trends among PWID regarding HIV risks have been seen within Vietnam and globally [36–38] demonstrating the potentially risky behaviors that PWID are engaging in. While certain practices that they are engaging in, like not sharing needles should further be promoted, their attitudes to, for example using condoms, need to be altered to further decrease HIV risk. Changing attitudes among this group of PWID should help to change attitudes and practices among the hidden PWID population and therefore reduce HIV risk behavior.

Most participants had utilized HIV testing and counselling services and 92.6% had had a HIV test. Of these only 17.4% had a positive result. This is significantly lower than previous other studies in Vietnam which have placed the HIV rate among PWID at closer to 30% [32, 38, 39] indicating that, in the mountainous areas, HIV may be well controlled. It should also be acknowledged however that this study deals with a specific cohort of PWID who are actively seeking health care services are therefore may engage in safer practices. Most patients identified that they used provincial level centers for HIV services rather than commune level. This could be a barrier to HIV risk prevention as patients may not be able to attend provincial centers easily or frequently. A preference among MMT users for integrated and decentralized services has been suggest by Tran et al. [40] while people from mountainous regions living far from services are also less likely to use them [33]. Given the mountainous population being examined, long travel times to MMT and HIV services may be a deterrent. For PWID registering for MMT to improve their KAP of HIV and to prevent HIV transmission among this vulnerable group, integration of MMT and HIV services may be necessary. This may also assist in decreasing the hidden drug using population and increasing the number of people receiving comprehensive care.

This study is a baseline assessment of a longitudinal follow-up of MMT patients who were taking MMT in newly established clinics in mountainous areas of Vietnam. Therefore, it has several implications for designing comprehensive health services and interventions in this at-risk group. Firstly, mass media targeting of community and individual counseling services for naïve patients could be beneficial in reducing transmission and risk behaviors and promoting HIV services access, utilization and outcomes among PWID in mountainous areas. With integrated HIV and MMT services, including counselling services, attitudes and practices could be altered to not only reduce the risk of those receiving MMT but to also to those who remain hidden drug users. Moreover, increased services could help to access those who currently do not engage due to geographical barriers.

This study has some limitations that should be acknowledged. This included the cross-sectional baseline design that limits the causal inference of KAP and HIV service uptake. In addition, the sample included only those accessing MMT services, and therefore, does not cover other at risk groups. This could introduce bias into the population selected as they may already have decreased risk behaviors as they engage freely with health services. Recall bias should also be considered a limitation of the study as data relied on participants self-reporting of drug use and HIV KAP. In addition, though being a rather reasonable choice given constraints of a study conducted in remote, under-developed area, the convenience sampling method adopted in this study may undermine the generalizability of our findings. Further studies on the topic with attempts to address these limitations, while also considering the inclusion of data on self-reported risk behaviors like injections and sexual behaviors, thus are being called for.

## Conclusions

As there is a lack of data for mountainous regions in Vietnam regarding both MMT and HIV, this study sheds significant light on the knowledge and HIV services access among PWID in disadvantaged settings. This then could inform the design and development of services and intervention for this group.

## Abbreviations

MMT: Methadone maintenance treatment
DALYs: Disability-Adjusted-Life Years
PWIDs: People who inject drugs
